# Supplementary material for: Genome sequencing of Plasmodium malariae identifies continental segregation and mutations associated with reduced pyrimethamine susceptibility
Source: Nat Commun. 2024 Dec 30;15:10779. doi: 10.1038/s41467-024-55102-3 (PMC11685946; doi:10.1038/s41467-024-55102-3)
Supplement: Supplementary file 1 — Supplementary Information [file 41467_2024_55102_MOESM1_ESM.pdf]

Supplementary figures

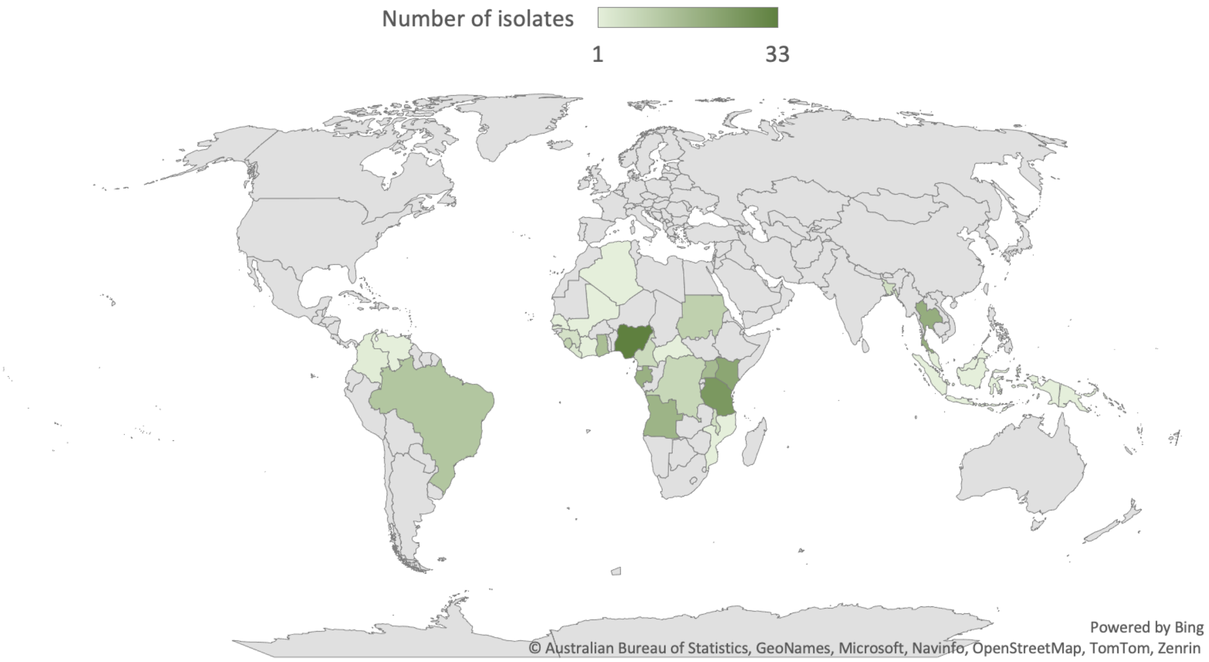

Supplementary Fig. SF1. Distribution, by country, of all *P. malariae* isolates with sequence data generated (novel and previously published) before filtering (N = 251). See **Supplementary Table ST2** for metadata of all isolates and accession numbers. Map was created using data from OpenStreetMap (openstreetmap.org) which is open data under license in the Open Data Commons Open Database License (ODbL) (opendatacommons.org) by the OpenStreetMap Foundation (OSMF) (https://www.openstreetmap.org/copyright).

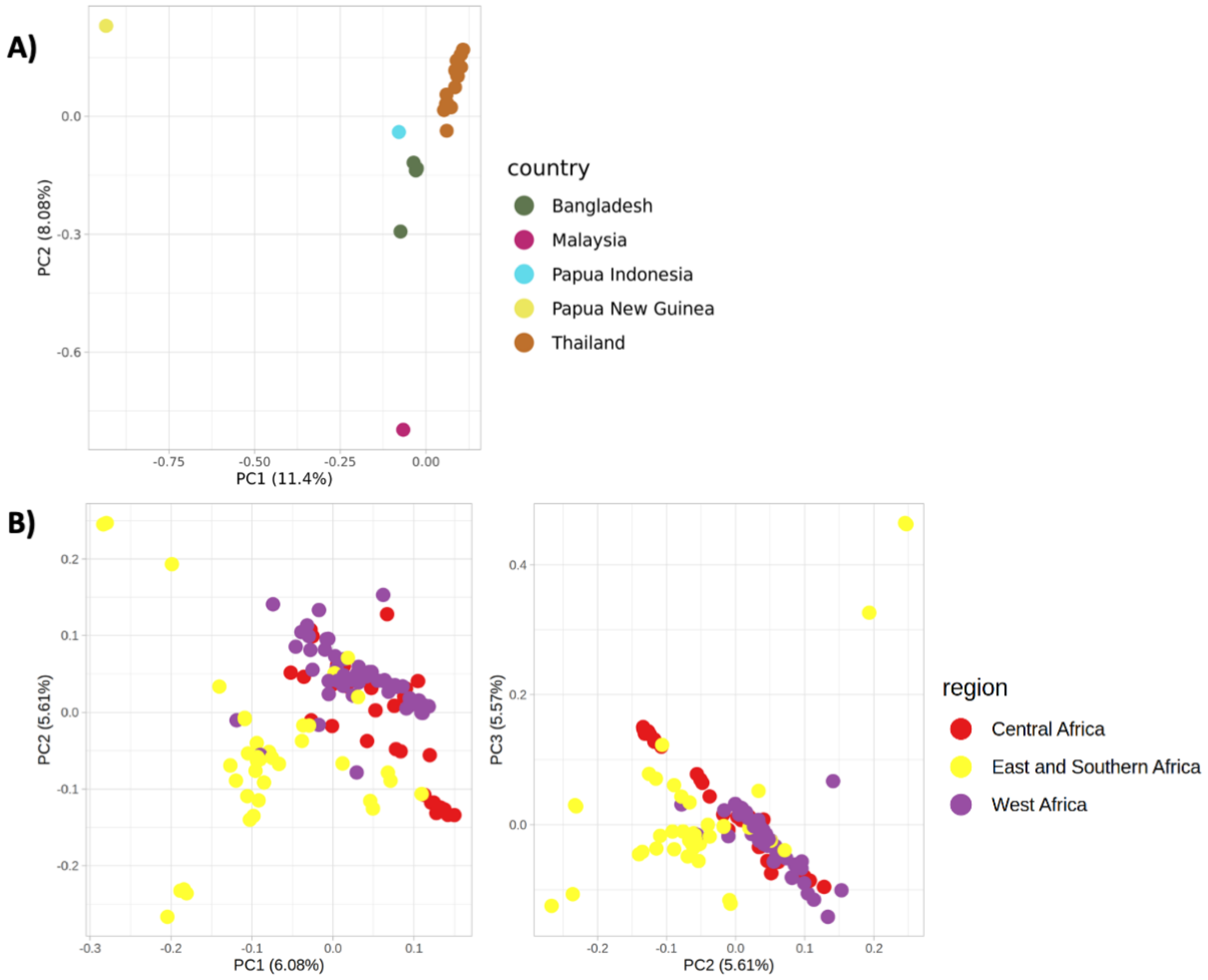

Supplementary Fig. SF2. Principal Component analysis of population structure. A) The two major axes (PC1 and PC2) differentiate 22 Asian isolates and an individual isolate from Oceania (Papua New Guinea) coloured by their country of origin. Each data point represents an individual isolate; **B)** The three major axes (PC1, PC2 and PC3) differentiate 128 *P. malariae* isolates obtained in Africa, coloured by the region of source (Central Africa, East and Southern Africa or West Africa). Each data point represents an individual isolate.

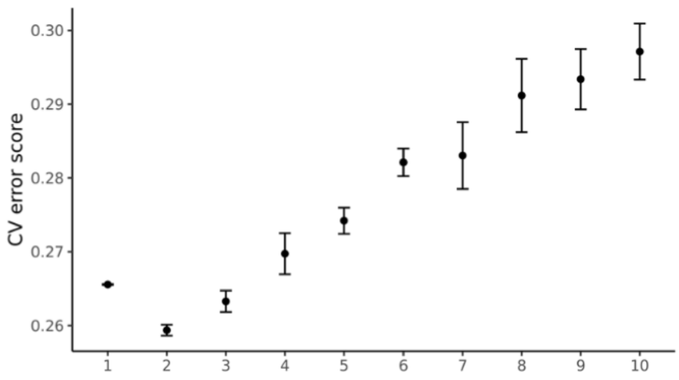

Supplementary Fig. SF3. Ancestral analysis. Estimation of number of ancestral populations amongst 141 isolates using ADMIXTURE software. ADMIXTURE was performed independently in triplicate for up to 10 populations to determine the cross-validation (CV) error score, and the average error scores were calculated. Individual data points on the graph represent the average error score, with error bars showing the range of error scores calculated over the three independent replicates. The lowest CV error score was obtained for 2 populations (average CV error = 0.259).

A)

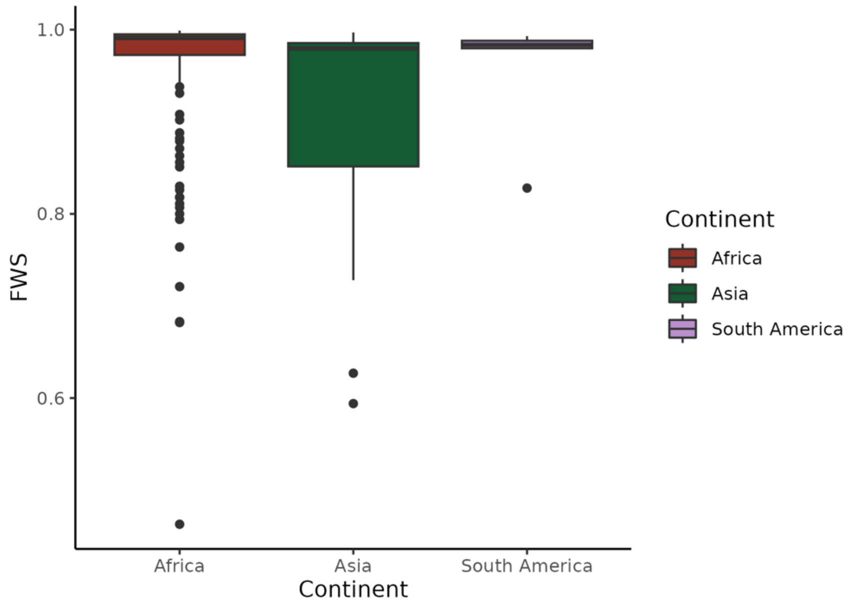

B)

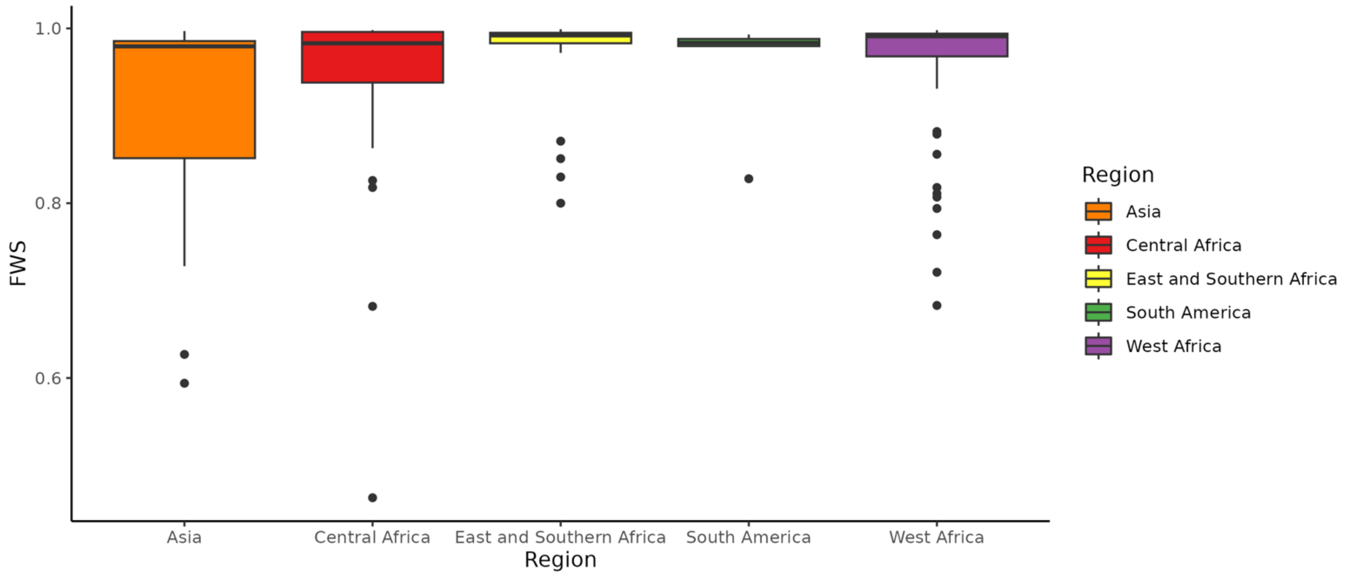

Supplementary Fig. SF4. Multiclonality in isolates in the filtered global database (n = 157). Multiclonality is calculated using F_{WS} scores through Moimix software. Boxplots demonstrate the range of clonality in isolates in each: **A)** Continent (Africa n = 128, Asia n = 22, South America n = 6), or **B)** Region (Asia n=6, Central Africa n = 29, East and Southern Africa n = 39, South America n = 6, West Africa n = 54). Boxes are coloured according to the origin of isolates, matching all other population genetics analyses. Samples were grouped by region to calculate F_{WS} scores, and the single isolate within Oceania has been removed from F_{WS} scoring due to small sample size. The mean (SD) F_{WS} are: Africa Central (0.939 (0.110)), Africa East and Southern (0.976 (0.047)), Africa West (0.968 (0.056)), Africa Continent (0.956 (0.081)), Asia (0.903 (0.129)), and South America (0.959 (0.065)).

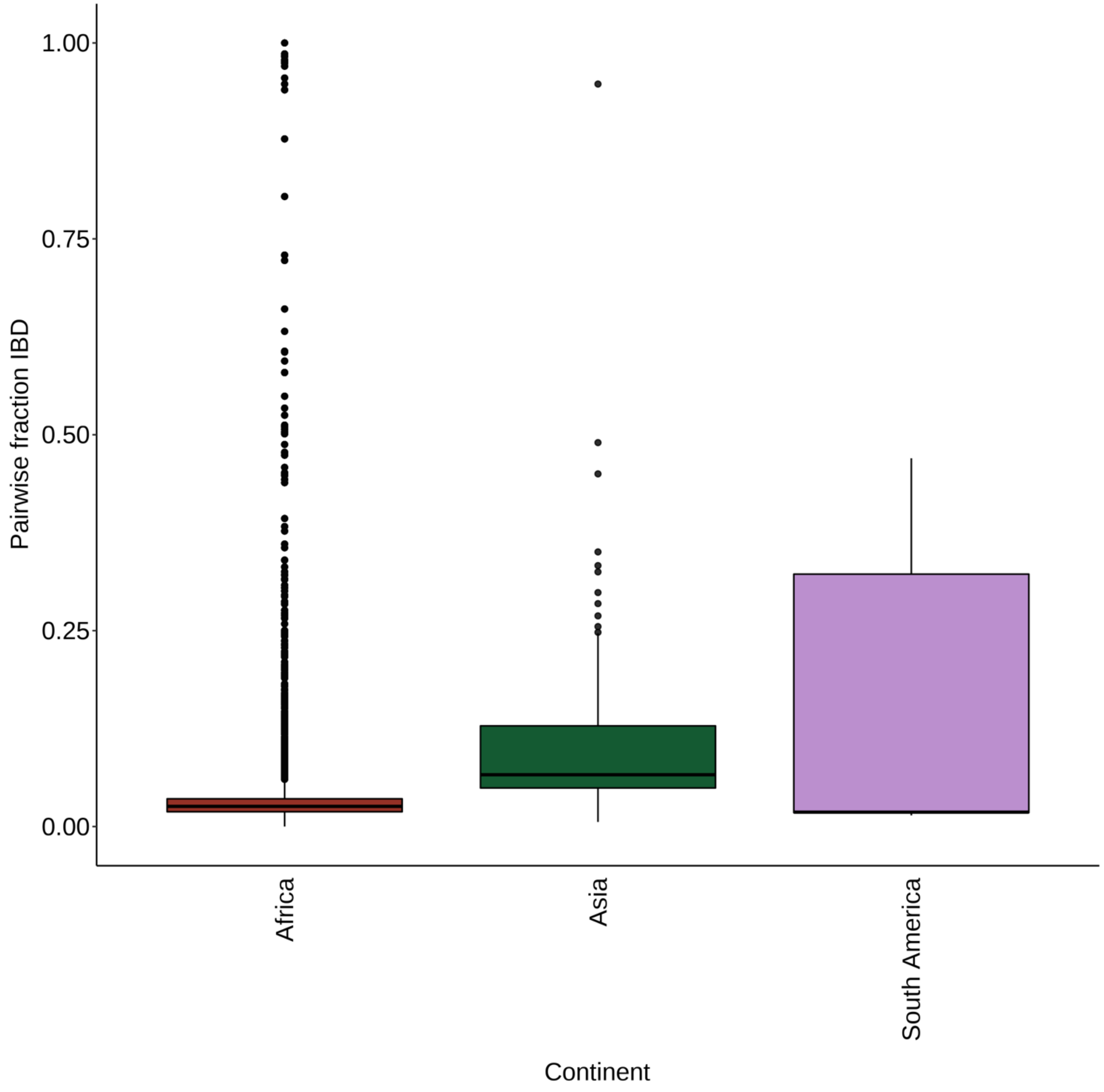

Supplementary Fig. SF5. Signals of homology within parasite populations in the global dataset determined using hmmIBD software. Identity by descent (IBD) was calculated for each continent group where there were > 5 monoclonal isolates (excluding Oceania). Monoclonality determined by F_{WS} score > 0.85, leaving a total of 136 monoclonal isolates (n: Africa 115, Asia 16, South America 5). Total IBD scores for all isolates in each continental group are visualised in boxplots.

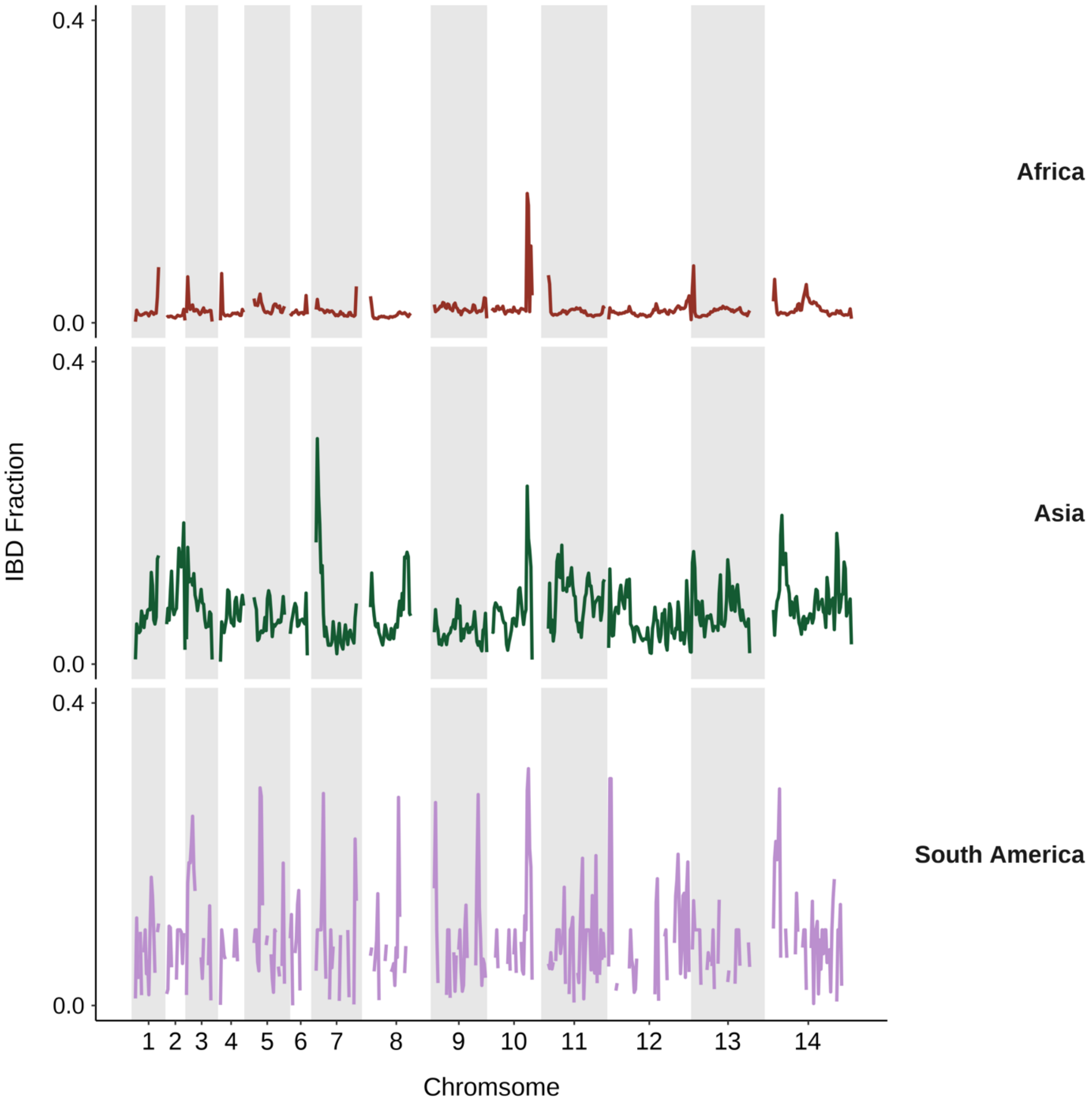

Supplementary Fig. SF6. Genome-wide signals of high identity by descent (IBD) across the three continents. Average identity by descent (IBD) scores amongst all monoclonal isolates (F_{WS} >0.85, n = 136) within each continent (n: Africa 115, Asia 16, South America 5) summarised across the length of the *P. malariae* genome using sliding windows of 10 kbp. IBD scores calculated using hmmIBD.

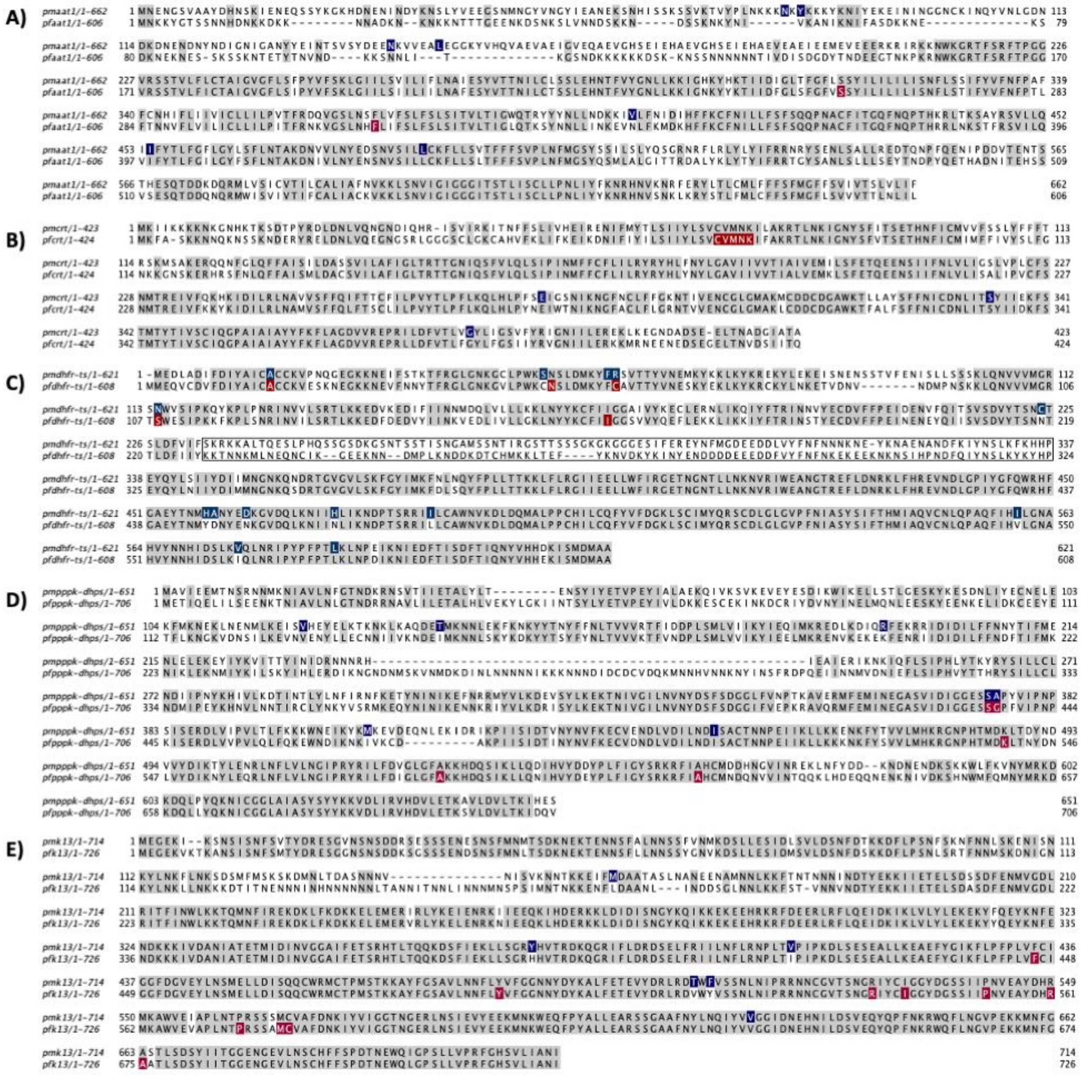

Supplementary Fig. SF7. Amino acid alignments to investigate relevance of SNPs found within orthologs of resistance-associated genes. Both the Pf3D7 and PmUG01 reference genome predicted protein sequences for each gene of interest were downloaded from PlasmoDB and aligned using Clustal Omega for: **A)** AAT1 (PF3D7_0629500 and PmUG01_11034100), **B)** CRT (PF3D7_07090000 and PmUG01_01020700), **C)** DHFR-TS (PF3D7_04172000 and PmUG01_05034700), with the linker region annotated by a black outlining box, **D)** PPPK-DHPS (PF3D7_08108000 and PmUG01_14045500), and **E)** Kelch13 (PF3D7_13437000 and PmUG01_120212000). Alignments were visualised in JalView, with conserved amino acids highlighted in grey, residues validated to be involved in resistance in *P. falciparum* in red, and residues found to be mutated in the *P. malariae* database in blue**.**

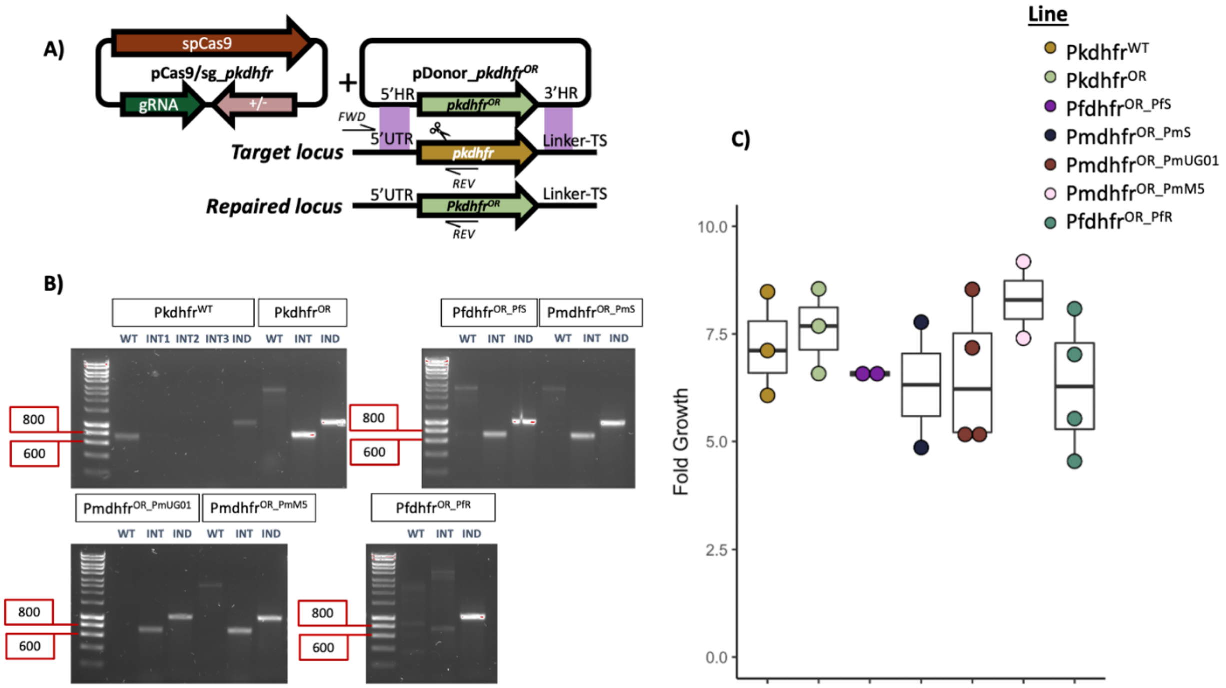

Supplementary Fig. SF8. Ortholog replacement of *pkdhfr*. A) Diagram outlining the strategy for genome editing of *P. knowlesi* parasites using CRISPR-Cas9 with a two-plasmid approach. One plasmid encoding the Cas9 endonuclease in addition to the guide RNA targeting Cas9 to the DHFR domain, and the other plasmid containing the donor DNA flanked by two 500 bp homology regions. The same guide plasmid (pCas9/sg_pkdhfr) was used for all 6 transfections, with 6 different donor plasmids for each individual ortholog replacement. Ortholog replacement occurred through homologous recombination after Cas9 induced double strand DNA break, with 500 bp 5' and 3' homology regions flanking either side of the replacement DHFR sequence (marked 5' HR and 3' HR). The locations of the primers used to confirm successful recombination are indicated with 'WT FWD' 'OR FWD' or 'REV'. The same FWD primer is used for all lines (olAI034), situated outside of the genetically modified locus within the *P. knowlesi* genome, and the REV primer is located within the new DHFR sequence after ortholog replacement, with a genus specific REV primer for *P. falciparum* (olAI039), *P. malariae* (olAI037), the recodonised *P. knowlesi* sequence (olAI036) and the parental *P. knowlesi* line (olAI035). **B)** Diagnostic PCRs of all parasite lines after transfection, clonal dilution, and DNA extraction to confirm parasites only harbour the modified locus. Three separate PCRs are completed for each line (WT - wild-type locus, olAI035 and olAI034; INT – integration locus, primers described above; IND – an independent PCR as a control targeting an unrelated locus within the *P. knowlesi* genome, primers ol75 and ol76). PCR primers and band sizes in **Supplementary Table ST11.** The parental parasite line *pkdhfrWT* was tested with all three integration primer sets (INT1 = *P. falciparum,* INT2 = *P. malariae*, and INT3 = *P. knowlesi* recodonised sequence). All primer sets were additionally tested with water as a negative control and showed no amplification. **C)** Fold growth of all parasite lines in human erythrocytes (Duffy positive) using one intraerythrocytic cycle of 27 hours. Fold growth was measured using a SYBR-Green I assay and flow cytometry, with all isolates having three technical replicates.
