# Supplementary material for: Genome sequencing of Plasmodium malariae identifies continental segregation and mutations associated with reduced pyrimethamine susceptibility
Source: Nat Commun. 2024 Dec 30;15:10779. doi: 10.1038/s41467-024-55102-3 (PMC11685946; doi:10.1038/s41467-024-55102-3)
Supplement: Supplementary file 3 — Description of Additional Supplementary Files [file 41467_2024_55102_MOESM3_ESM.pdf]

Description of Additional Supplementary Files

File Name: Supplementary Data 1
Description: Isolates in *P. malariae* genomic database

File Name: Supplementary Data 2
Description: Metadata for all isolates included in the genomics database.

File Name: Supplementary Data 3
Description: Metadata of isolates in the filtered genomics database.

File Name: Supplementary Data 4
Description: Differentiating SNPs with an FST 1 when comparing African and Asian isolates.

File Name: Supplementary Data 5
Description: Distribution of pairwise IBD scores between isolates within each continent.

File Name: Supplementary Data 6
Description: Genomic segments with IBD hits per continent for all monocloncal isolates.

File Name: Supplementary Data 7
Description: Within continent iHS scores for monoclonal isoaltes in Africa (n = 155) and Asia (n = 16).

File Name: Supplementary Data 8
Description: Differential signals of positive selection (Rsb score) when comparing monocloncal isoaltes from Africa (n = 155) and Asia (n 16).

File Name: Supplementary Data 9
Description: SNPs found in putative drug resistance markers in the *P. malariae* genomics database.

File Name: Supplementary Data 10
Description: Global revalence of non-synonymous SNPs found in putative drug resistance markers in the *P. malariae* genomics database.

File Name: Supplementary Data 11
Description: Prevalence of DHFR mutations within regions in Africa.

File Name: Supplementary Data 12
Description: Primer sequences used in this study along with expected DNA fragment sizes.
